# Thickness-Induced Metal-Insulator Transition in Sb-doped SnO_2_ Ultrathin Films: The Role of Quantum Confinement

**DOI:** 10.1038/srep17424

**Published:** 2015-11-30

**Authors:** Chang Ke, Weiguang Zhu, Zheng Zhang, Eng Soon Tok, Bo Ling, Jisheng Pan

**Affiliations:** 1 Microelectronics Centre, School of Electrical and Electronic Engineering, Nanyang Technological University, Nanyang Avenue, Singapore 639798; 2 Institute of Materials Research & Engineering, A*STAR (Agency for Science, Technology and Research), 3 Research Link, Singapore 117602; 3 Department of Physics, National University of Singapore, Science Drive 3, Singapore 119260.

## Abstract

A thickness induced metal-insulator transition (MIT) was firstly observed in Sb-doped SnO_2_ (SnO_2_:Sb) epitaxial ultrathin films deposited on 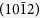 sapphire substrates by pulsed laser deposition. Both electrical and spectroscopic studies provide clear evidence of a critical thickness for the metallic conductivity in SnO_2_:Sb thin films and the oxidation state transition of the impurity element Sb. With the shrinkage of film thickness, the broadening of the energy band gap as well as the enhancement of the impurity activation energy was studied and attributed to the quantum confinement effect. Based on the scenario of impurity level pinning and band gap broadening in quantum confined nanostructures, we proposed a generalized energy diagram to understand the thickness induced MIT in the SnO_2_:Sb system.

Doping of semiconducting materials with shallow impurities (donor- or acceptor-types) determines to a large extend the electronic properties of the host. It’s generally accepted that the electronic properties will be significantly affected when the size of crystal is close to its effective Bohr radius (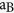). Benefiting from modern techniques, the device miniaturization is approaching nanometer scale, and consequently the role of quantum confinement in the electronic states of shallow impurities becomes significant. As a fundamental issue in condensed matter physics, the size dependence of metal-insulator transition (MIT) has been well documented from metals to intrinsic metallic oxides, such as Au, Al, V_2_O_3_, VO_2_ and SrRuO_3_^1^,^2^,^3^,^4^,^5^,^6^,^7^. However, few experimental work has shed light on the quantum confinement induced MIT in the impurity doped metal oxides, which has been predicted by theoretical calculations^8^,^9^. Sb-doped SnO_2_ (SnO_2_:Sb) has been a system of interest due to its ability to simultaneously combine a high optical transparency with a low sheet resistance^10^. By increasing the Sb concentration above the Mott critical density (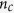), metallic electrical transport behavior can be achieved in SnO_2_, which favors its applications as a transparent conductive oxide (TCO)^11^. At present, however, as far as quantum confinement effect is concerned, it is not clear when the macroscopic laws that give the metallic conductivity in SnO_2_:Sb will fail (i.e., the critical scale for transition from metal to insulator) ? In the same context, it is also not clear how will the electronic properties of shallow impurity will similarly be affected ? Addressing these questions is not only important from a fundamental point of view, but also beneficial to the design of miniaturized devices.

In this work, we conducted electrical and spectroscopic studies on a series of SnO_2_:Sb epitaxial thin films deposited on 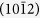 sapphire substrates. By precisely controlling the film thickness ranging from bulk (~200 nm) to quantum confinement scale (~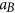), a thickness induced MIT was observed from the temperature-dependent measurements on the electrical transport behavior. Further evidence of this transition was also provided by X-ray photoelectron spectroscopy (XPS) characterizations. Analysis of the temperature-dependent resistivity behaviors in conjunction with the XPS core-level spectra yielded conclusions that the quantum-confined SnO_2_:Sb ultrathin film (i) the impurity activation energy (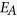) was enhanced, and (ii) the oxidation state of Sb changed from Sb(V) to Sb(III). Consequently, the MIT in these SnO_2_:Sb thin films emerged as the critical thickness was reached. A generalized energy diagram, based on the scenario of energetic impurity level pinning and energy band gap broadening in the doped semiconductor nanostructures, was proposed to understand the MIT in the SnO_2_:Sb system.

## Results and Discussion

**Figure 1:**
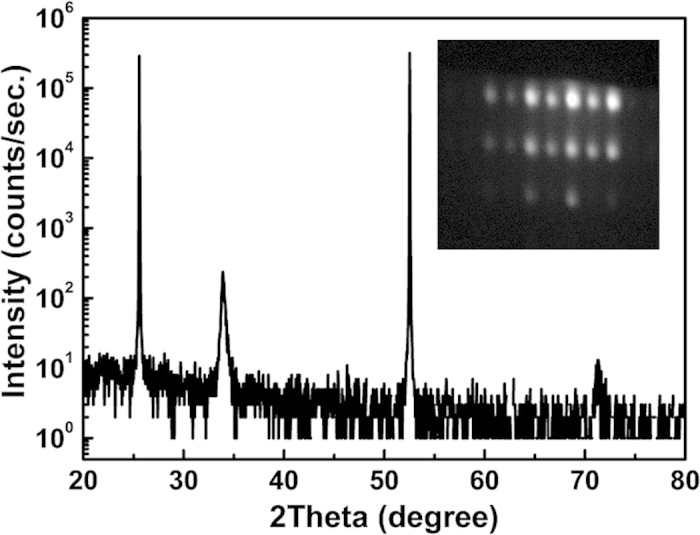
A typical XRD pattern of SnO_2_:Sb film on 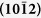 sapphire substrate. The inset shows the *in-situ* RHEED pattern of the as-deposited film.

The growth process of SnO_2_:Sb films on sapphire 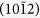 was *in-situ* monitored by the reflection high energy electron diffraction (RHEED). Similar RHEED patterns were obtained for all films. The inset picture in Fig. 1 gives a typical RHEED image of sample B with a thickness of 31.3 nm. The well-defined RHEED pattern indicates that the film is in single crystalline with Stranski-Krastanov growth mode, which is caused by the large lattice mismatch between the film and the substrate^12^. The phase structure was further investigated by HR-XRD. Figure 1 gives the 2θ-ω scan result of sample B, in which pure (101) orientation was achieved with no other detectable foreign phase or other orientated grains. This suggests that the SnO_2_:Sb thin film was epitaxially deposited with the out of plane epitaxial relationship of 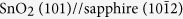. The surface morphologies of the SnO_2_:Sb films were taken (see Figure S1 in Supplementary Information), from which continuous and smooth surfaces were revealed.

**Figure 2:**
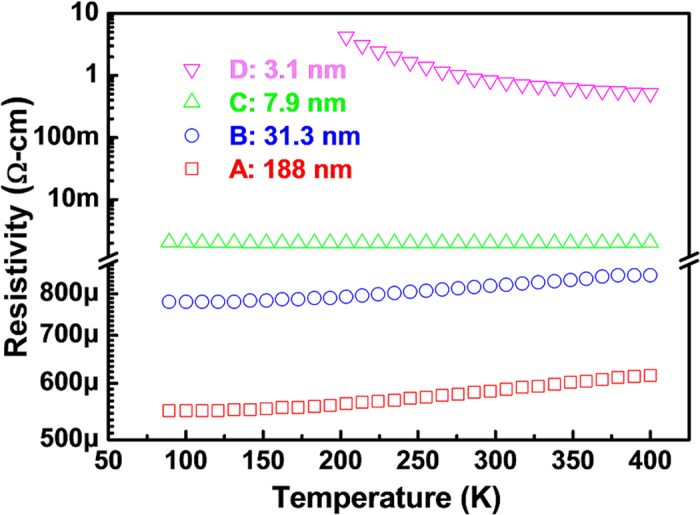


The in-plane electrical transport properties of the films were measured as a function of temperature ranging from 90 to 400 K. Figure 2 shows the plot of the temperature-dependent resistivity of each film. From these results, it can be seen that the resistivity of relatively thicker films (samples A and B) reduces as the temperature decreases, which suggests a metallic behavior. By contrast, semiconducting behavior (insulator ground state) was observed in the ultrathin film (sample D) with a thickness of 3.1 nm, for which the resistivity increases monotonically with the decrease of the temperature. As for the sample C, which possesses the thickness between those of samples B and D, its resistivity shows a very week dependence on the temperature. It is clear that a MIT has been induced by varying the SnO_2_:Sb film thickness from bulk value to nanoscale, and the critical thickness for this MIT should be around 7.9 nm.

**Figure 3:**
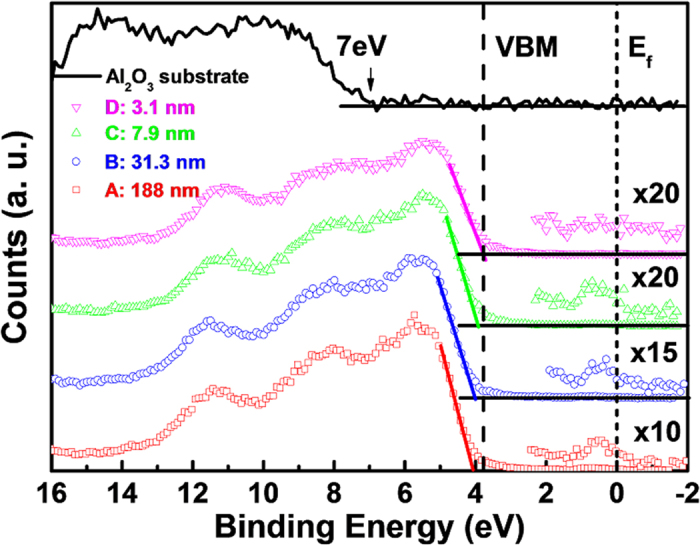
XPS results of the SnO_2_:Sb films with different thicknesses in the binding energy range from −2 to 16 eV. For comparison, the valence band spectrum of pure Al_2_O_3_ substrate is also presented. The substrate effect on the valence maximum denotation and conduction band electron measurement of the SnO_2_:Sb can be neglected due to little contribution from Al_2_O_3_ below 7 eV.

In order to get a better insight into this thickness induced MIT, XPS was employed to investigate the electronic structure of the SnO_2_:Sb thin films. Figure 3 presents the photoemission spectra of four samples in the binding energy ranging from −2 to 16 eV. This region encompasses the conduction band, the bulk band gap, and the valence band. By zooming into the zero-binding-energy region, a prominent Fermi-Dirac-like cutoff associated with electrons in the conduction band is observed in samples A to C, which confirms the metallic property of these SnO_2_:Sb films at room temperature. These results are consistent with the resistivity measurements shown in Fig. 2. The electron occupation in the conduction band vanished as the film thickness scaled down to 3.1 nm in sample D. The absence of a Fermi-edge cutoff clearly indicates its insulator nature. According to these XPS results, the thickness induced MIT in SnO_2_:Sb thin films is further confirmed. In addition, the electron emission signal from conduction band region was found to gradually reduce as the film thickness decreased (Fig. 3). This occupation decrement implies a thickness induced reduction of free electron concentration. To verify the above behavior, the room-temperature electron concentrations (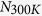) were measured by Hall effect as summarized in Table 1, in which a clear shrinkage of electron concentration is revealed. In addition, the valence band maximum (VBM) of each sample can be quantified by extrapolating the leading edge of valence band (VB) spectra in Fig. 3 to intersect with the background base line. This procedure has been used to determine the VBM position of conventional semiconductor materials^13^,^14^. The resolution of extrapolation of our samples is found to be ±0.05 eV. The VBM values are summarized in the Table 1. It is clear that the VBM shows a red shift to lower binding energy side with the decrease of film thickness (i.e. the shrinkage of electron concentration shown in Fig. 3 and Table 1). This VBM shift can be interpreted by the Burstein-Moss (BM) effect originated from the filling of the conduction band^15^. Considering a free-electron profile of Sn 5 s conduction band for SnO_2_, the magnitude of BM induced VBM shifts in XPS spectra is directly related to the occupied conduction bandwidth, which can be calculated by 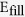
13:


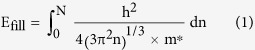


Here, 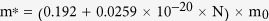 gives the carrier concentration dependent electron effective mass. 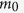
and 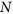 represent the electron mass and electron concentration respectively. The parameters for 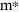 were obtained from the published data^13^,^16^. By using equation (1) and electron concentrations obtained from Hall measurements, the values of 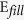 were calculated and shown in the Table 1. It can be seen that the higher electron concentration, the wider the occupied conduction band. Accordingly, the VBM edge is pushed to higher energy side in the XPS spectra, which further confirms the self-consistency of the results in Table 1.

From the above-mentioned temperature-dependent resistivity results and the XPS VB spectra, the thickness induced MIT in metallic SnO_2_:Sb thin films was confirmed. However, questions arise such as: Where have the free electrons gone ? What is the origin of this MIT behavior in these thickness varied SnO_2_:Sb thin films ? To address these questions, the role of Sb in SnO_2_ lattice should be investigated, considering that the metallic properties of SnO_2_:Sb originate from the overlapping between the donor level (Sb(V)) and the SnO_2_ conduction band. From the real space point of view, metallic SnO_2_:Sb can only be achieved as long as the effective Sb(V) concentration is higher than the 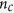. The doping concentration of Sb in our SnO_2_:Sb target (around 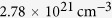) is much higher than the theoretical value of 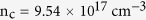 for SnO_2_^17^. In spite of the stoichiometry deviation between the film and target, the metallic properties indeed have been observed in the relatively thicker SnO_2_:Sb films. However, Sb is a multivalent element with two common oxidation states of Sb(V) and Sb(III). In SnO_2_, only the Sb(V) acts as donor while the Sb(III) tends to be acceptor compensating the free electrons contributed by Sb(V). Therefore, the oxidation state of Sb should directly relate to the carrier concentration and electrical transport behavior in our SnO_2_:Sb thin films. The coexistence of Sb(V) and Sb(III) in SnO_2_:Sb films has been previously reported^18^. In order to investigate the oxidation state of Sb, high-resolution Sb 3d_3/2_ core-level spectra of these thickness-varied SnO_2_:Sb films have been measured.

**Figure 4:**
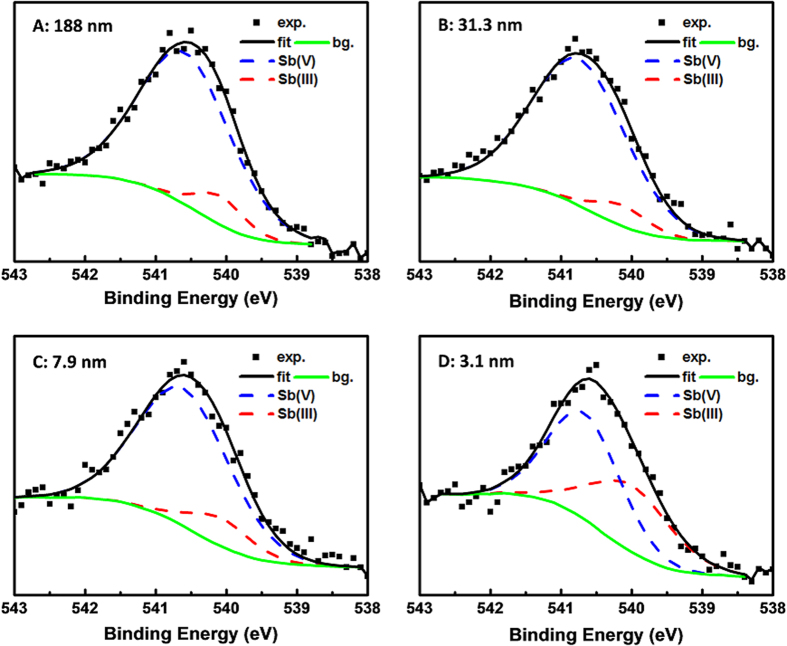
Core-level Sb 3d_3/2_ XPS spectra of SnO_2_:Sb films (filled squares) with different thicknesses: (**A**) 188.0 nm, (**B**) 31.3 nm, (**C**) 7.9 nm, and (**D**) 3.1 nm. The spectra were fitted to a Shirley background (green line) together with the Voigt profiles for combined Sb(V) and plasmon satellite (blue dash lines) and Sb(III) (red dash line). The fitting curves (black solid line) are seen to match well with the experimental data points. The denotation exp., fit., and bg. represent experimental data, fitting curve, and back ground, respectively.

As shown in Fig. 4, the fitting of the Sb 3d_3/2_ core lines into two Voigt components gives a good description of the overall core line shape in which the lower binding energy peak represents the Sb(III). However, the higher binding energy component is complicated, since it may be the combination of main Sb(V) (screened final state) and plasmon satellite (unscreened final state) peaks. Due to the relatively low doping concentration of Sb in the samples and also the very thin film thickness, the signal-to-noise ratio of the Sb 3d_3/2_ spectra is too low to separate the peaks. Accordingly, a broad high binding energy peak is used to fit the spectra. The plasmon satellite peak in the XPS core lineshapes in degenerately doped semiconductors has been well documented^13^,^19^,^20^. It usually appears as a shoulder at the higher energy side of the core line. In addition, according to Egdell *et al.*’s works, the plasmon satellite peak is strongly correlated to the electron concentration, i.e. the lower of electron concentration, the weaker of plasmon satellite peak^13^. Since the sample D is non-metallic, which means the electron concentration is low, its high binding energy peak does not have unscreened component. Accordingly, the observation of a narrower high binding energy peak in sample D can be explained.

To have a better view on how does the Sb change in the four SnO_2_:Sb samples, the variations of Sb/Sn and Sb(III)/Sn ratios are analyzed and shown in the Table 1. First of all, it can be seen that the Sb/Sn ratio does not show any trend as the film thickness reduces from 188.0 nm to 3.1 nm, which means the measured Sb doping concentrations in the films are independent from the thickness. This is consistent with that all the four samples were fabricated at the same conditions. As for the Sb(III)/Sn ratio, it is clear that the thinner the SnO_2_:Sb film is, the higher concentration of Sb(III) exists in the film. This oxidation state transition of Sb will cause the reduction of electron concentration as film thickness shrinks, and consequently a MIT occurs when the effective Sb(V) density is lower than the Mott critical criteria. However, regarding the Sb doping in SnO_2_, one issue, i.e. segregation of Sb at the surface, should be considered, since the segregated Sb traps two electrons as Sb(III). According to the literatures, the Sb surface segregation has been well studied by Egdell *et al.*^21^,^22^ and Szczuko *et al.*^18^ Both of them found that the Sb segregation is restricted to the topmost ionic layer of SnO_2_, i.e. a single surface plane of tin ions is replaced by Sb. Accordingly, the Sb segregation layer is only a very small portion of the film even for the thinnest sample in this study. The electrical transport behavior and electronic band structure discussed in this work are the combination effect of the surface and bulk of the films. Considering the Sb segregation only exists in the topmost layer, its contribution to the MIT is negligible in this study. In addition, all the samples in this study should have similar Sb segregation layer on the top, since they were prepared through the exact same process. This can be supported by the sample topography revealed by AFM shown in the Fig. S1 in Supplementary Information. Accordingly, it can be concluded that the thickness induced MIT observed in this study is unlikely related to the Sb surface segregation. Although Sb segregation at the topmost ionic layer has small contribution to the electrical transport property, i.e. MIT itself, the XPS core-level measurements, i.e. Sb oxidation state analysis could be significantly affected due to the surface sensitive nature of XPS technology. More specifically, the increase of Sb(III) in the ultra-thin samples could be due to the increasing importance of segregation, since some of the samples could be thinner than the XPS probing depth. To address this issue, the XPS survey spectra of the four samples are collected and shown in the Fig. S2 in Supplementary Information. The XPS probing depth of our samples is found to be between 7.9 nm and 3.1 nm. Accordingly, for the XPS spectra of sample A, B and C, the contributions from the surface layer should be the same. However, based on the Table 1, the sample C has a higher Sb(III)/Sn ratio than the sample B and A. This result implies that the increase of Sn(III) could not be attributed to the surface layer contribution variation. In addition, as for the sample D, its thickness is less than the XPS probing depth, which means the surface contribution in sample D is greater than the other three samples. This makes the analysis to the Sb(III) variation in sample D more complicated. However, if we assume the XPS probing depth is 6 nm, the surface contribution in sample D can be estimated to be 2 times of the other samples, since the sample D has a thickness of 3.1 nm. Referring to the Table 1, the Sb(III) concentration in sample D is 2.7, 3.3 and 3.4 times of sample C, B and A, respectively. This estimation shows that the sample D still has more Sb(III) than the other samples, although the surface contribution is higher in sample D. As a result, It can be concluded that the thickness dependent Sb(III) concentration variation is not simply because of surface layer contribution variation.

**Figure 5:**
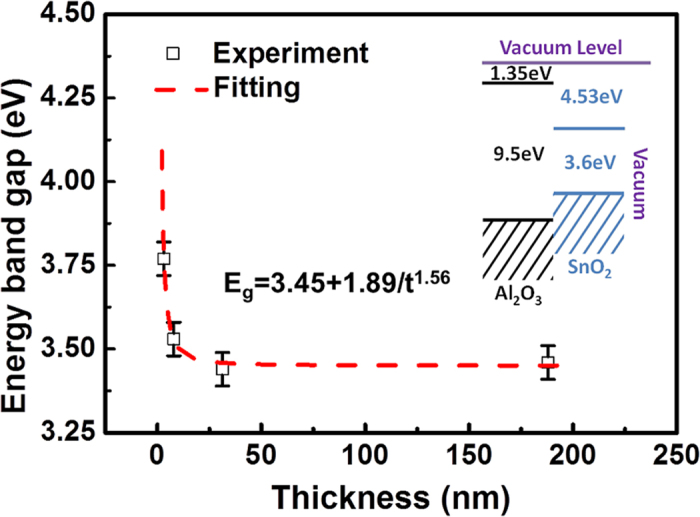
The energy band gap of different thickness SnO_2_:Sb thin films as extracted from the XPS valence band spectra. The dash line shows the least-squares fit to a power-law function: 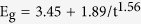, from which a remarkable quantum confinement effect is revealed.

Depending on the above analyses of Sb 3d_3/2_ core-level spectra, Sb(III) is preferred for the ultrathin SnO_2_:Sb film, and this oxidation state transition gives rise to the MIT. To understand this film thickness related phenomena, the quantum confinement effect should be considered as the film thickness approaches its Bohr radius (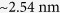)^17^. Referring to the energy band diagram of the SnO_2_:Sb/Al_2_O_3_ structure, as shown in the inset of Fig. 5, the electrons in the films are confined in the potential well formed by Al_2_O_3_ substrate and vacuum. It’s generally accepted that the energy band gap (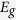) will be significantly broadened when the electrons are squeezed. To explore the thickness effect on the electronic structure of the SnO_2_:Sb films, we extracted the values of 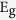 from the XPS spectra (shown in Fig. 3) by subtracting the conduction band filling (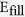) from the VBM (see the part 3 and Fig. S3 in Supplementary Information). In addition to 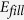, the band gap renormalization should also be considered to calculate the fundamental 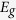, since all the SnO_2_ films were highly doped with Sb. Bandgap renormalization caused by electron-electron interaction and electron-impurity interaction has been extensively studied in multiple material systems, e.g. CdO, ZnO and SnO_2_^13^,^19^,^23^,^24^,^25^,^26^,^27^. Based on Egell *et al.*’s^13^ and Walsh *et al.*’s^27^ works the total band gap narrowing (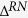) in our samples can be estimated as: 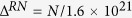, where N is the electron concentration. Accordingly, the fundamental 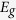 can be calculated by equation, 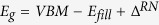. A prominent increase of 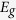 with the decrease of film thickness was observed, as shown as in Table 1 and Fig. 5. By analytic fitting to the thickness-dependent 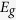 shown in Fig. 5, the 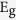 shift scales as 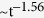, 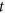 here denotes the thickness of thin film. The magnitude of the scaling power is found to have a similar value with the cases of SnO_2_ quantum wires (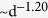) and quantum dots (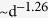) predicted by first principles calculations, where 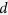 stands for the radius^28^. Besides the quantum confinement effect induced band structure change, the strain effect on band structure should also be considered, since it usually exists in heteroepitaxial thin films. Recently Zhang *et al.* reported that the tensile strain presents in a series of In_2_O_3_ epitaxial thin films with thickness ranging from 35 to 420 nm, due to the 1.6% lattice mismatch between the In_2_O_3_ film and YSZ substrate. In addition, a 0.08 eV red shift to the band gap of In_2_O_3_ films was found to be caused by the thickness modulated tensile strain^29^. Accordingly, the strain effect in our samples should also be considered for band gap analysis. It has been well established that the residual strain in an epitaxial film is highly dependent on the lattice mismatch between the film and substrate. Specifically to (101)-SnO_2_ film on 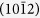-Al_2_O_3_ substrate, the lattice mismatch along the SnO_2_ 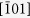 direction, which is parallel to the 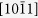 direction of Al_2_O_3_, is very high, i.e. 11.45%^30^. With such a big lattice mismatch, the substrate induced strain will be relaxed through a series of quasiperiodic misfit dislocations running along the 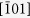 direction of SnO_2_^30^. In addition, based on Matthews-Blakeslee theory^31^,^32^, the critical thickness for (101)-SnO_2_ epitaxial film to preserve perfect lattice matching with 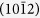 Al_2_O_3_ substrate can be estimated to be less than one SnO_2_ monolayer, i.e. 0.26 nm. This means the lattice relaxation happens in the first one or two layers of SnO_2_ from the SnO_2_-Al_2_O_3_ interface. The thinnest film in this work is 3.1 nm-thick, which is considerably greater than the critical thickness. Accordingly we believe the strain in the samples in this work has been relaxed. To experimentally study the strain, symmetric 2θ-ω XRD scans across the Al_2_O_3_ 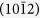, SnO_2_ (101) and Al_2_O_3_ 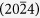 reflections for SnO_2_:Sb films with different thicknesses were collected and shown in the Fig. S4. Based on the SnO_2_ (101) reflection positions and calculated out-of-plane lattice distances in the Table S1, it can be seen that the film thickness variation does not affect the lattice parameters. This confirms the strain in the samples has been relaxed. As a result, the strain induced bandgap shift is insignificant to this study.

**Figure 6:**
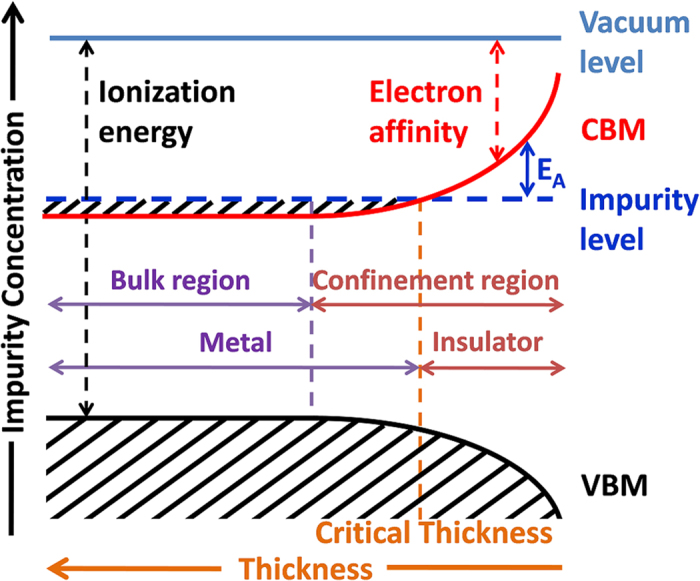


**Figure 7:**
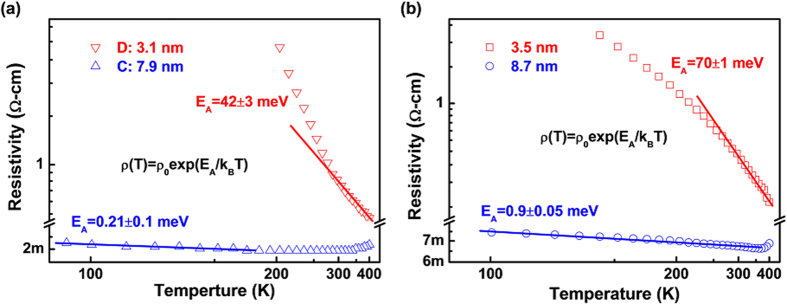
The activation energies of Sb in (**a**) SnO_2_:Sb10 films with thickness of 3.1 nm, 7.9 nm and (**b**) SnO_2_:Sb1 films with thickness of 3.5 nm, 8.7 nm.

More specifically, the quantum confinement induced 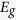 broadening can be attributed to two distinct effects: the increase of ionization energy and the decrease of electron affinity. This size induced electronic structure variation has been intensively studied in the pure IV, III-V, II-VI and IV-VI semiconductor nanostructures^28^,^33^,^34^,^35^,^36^,^37^,^38^,^39^. As such, the influence of quantum confinement effect on the physical properties of impurity doped semiconductor nanostructures is expected. Based on the first principles calculations, as the nanocrystal size decreases, (i) the impurity activation energy is enhanced^40^,^41^,^42^, (ii) effective Bohr radius is squeezed^8^,^41^,^43^, (iii) impurity wavefunction becomes more localized^43^,^44^,^45^, (iv) a valence state transition for the impurity is driven^9^, and (v) the energetic level of impurity is pinned referring to the vacuum level^8^. Based on the above analyses, a generalized energy diagram in the parameter space of film thickness and Sb doping concentration, for the impact of quantum confinement on our thickness-varied SnO_2_:Sb thin films was deduced and shown in Fig. 6. In this diagram, the ionization energy increases and the electron affinity decreases with the reduction of film thickness within the confinement region. At the same time, the impurity level is independent of the film thickness, which means it is pinned relative to the vacuum level. On the other hand, the position of the impurity level relative to the conduction band minimum (CBM) which is defined as activation energy (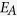) can be controlled by the doping concentration (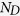). In the bulk region, the value of 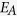 is given by the following equation^46^,^47^:


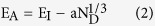


where 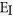 is the ionization energy for an isolated impurity center. In our SnO_2_:Sb thin films, the Sb-induced impurity level is higher than the conduction band of SnO_2_ when the film thickness is relatively larger (metal region in Fig. 6) and offers metallic conductivity. As the film thickness reduces, the electron affinity decreases while the impurity level is pinned, and consequently causes a weaker overlap between the conduction band and the impurity level which leads to a lower electron concentration. This free electron concentration reduction has also been confirmed in the Hall measurement results and XPS valence band spectra. With the further shrinkage of the film thickness, the CBM meets the impurity level at a critical thickness where a MIT happens. When the film becomes even thinner, the CBM surpasses the impurity level and the 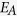 of Sb increases accordingly as shown in Fig. 6. To verify this prediction, we fit the resistivity data for sample D with an Arrhenius conduction model, in which the electrical resistivity is given by:


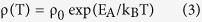


where 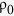 is the residual electrical resistivity and 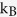 is Boltzmann constant. For sample C, the 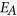 is extracted from data below 150 K in which a semiconducting behavior was observed. Fig. 7(a) gives the fitting results showing that 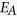 for samples C and D are 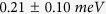 and 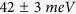, respectively. Indeed, a prominent 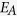 enhancement in the thinner film is observed, which is consistent with Fig. 6. Based on equation (2), with the decrease of doping concentration the impurity level will move downward relatively to the CBM as well as the vacuum level in the bulk region. Referring to Fig. 6, this downward shift of impurity level will lead to a higher critical thickness for the size induced MIT. Experimental implementation for this prediction has been done and demonstrated in Fig. 7(b). Similar to the 10% Sb doped SnO_2_ (SnO_2_:Sb10), a series of 1% Sb doped SnO_2_ (SnO_2_:Sb1) thin films were fabricated with the same parameters and characterized by Hall measurement. The deposition rate has been recalibrated as 0.58 Å per laser ablation for the new target of SnO_2_:Sb1. Figure 7(b) shows the temperature dependent resistivity of SnO_2_:Sb1 thin films with the critical thickness found to be around 8.7 nm, which is larger than that of SnO_2_:Sb10. The 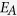 for this 8.7 nm thick SnO_2_:Sb1 thin film is calculated to be 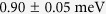. This value is large that the sample C with thickness of 7.9 nm and 10% Sb, which implies that the 8.7 nm thick SnO_2_:Sb1 thin film is “more insulating” than the 7.9 nm thick SnO_2_:Sb10 thin film. For the thinner SnO_2_:Sb1 film with thickness of 3.5 nm, its insulator nature is confirmed and the E_A_ is 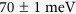. Compared with that of sample D which has 10% Sb and thickness of 3.1 nm, the 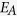 of Sb in the SnO_2_:Sb1 thin film increased substantially, which is consistent with the conclusions deduced from Fig. 6. At present, we look back to the previous analysis of the Sb 3d_3/2_ core-level spectra shown in Fig. 4, the observed oxidation state transition from Sb(V) to Sb(III) can be understood by the enhancement of the activation energy (electron binding energy) and the strong self-trapping of electrons by the Sb sites. Hence, both the experimental results and theoretical analysis indicate a quantum confinement induced MIT in the SnO_2_:Sb films and the existence of operational size limit for its microelectronic applications.

## Experimental Procedures

A pulsed laser deposition (PLD) system was employed to deposit the thin films. Energy density of the laser beam and laser ablation rate were fixed at 1.5 J/cm^2^ and 1 Hz, respectively. A homemade 10% Sb doped SnO_2_ ceramic target was prepared via standard solid state reaction process. Flat and uniform sapphire 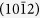 substrates with surface atomic steps (see atomic force microscopy (AFM) image in Fig. S5 for Supporting Information) were achieved with the technique described by M. Yoshimoto^48^. Four SnO_2_:Sb thin film samples with thicknesses of 188.0 nm, 31.3 nm, 7.9 nm and 3.1 nm (labeled as A, B, C and D, respectively) were fabricated on those sapphire substrates by varying the laser pulse counts at a fixed substrate temperature (T_sub_ = 700 °C) and under a constant oxygen partial pressure (P_oxy_ = 100 mTorr). The thin films were deposited at a growth rate of 0.52 Å per laser ablation as deduced from the thickness calibration of the thickest SnO_2_:Sb film (188.0 nm) using spectroscopic ellipsometry (J.A. Woollam, VASE). The reflection high energy electron diffraction (RHEED) was operated in anti-Bragg condition using 20 keV electron beam at a grazing incidence angle of 1°–3° toward substrate surface. Phase structural characterization was carried out by high resolution X-ray diffraction (HR-XRD, Philips X’pert MRD). The electrical transport properties, namely, carrier concentration n, dc resistivity ρ, and its temperature dependence were measured in Van der Pauw configuration using Hall measurement system (Bio Rad HL5500). Considering the gas-sensitive nature of SnO_2_, the chamber of Hall measurement was purged with nitrogen and evacuated in order to minimize the influence of ambient gas. XPS measurements were carried out using a VG ESCALAB 200i-XL system equipped with a monochromatic Al Kα (1486.6 eV) X-ray source, to obtain the core-level and valence band spectra of all the samples. Binding energy calibration was performed using gold (Au), silver (Ag), and copper (Cu) standard samples by setting the Au 4*f*_7/2_, Ag 3*d*_5/2_, and Cu 2*p*_3/2_ peaks at binding energies of 83.96 ± 0.02 eV, 368.21 ± 0.02 eV, and 932.62 ± 0.02 eV, respectively. The Fermi edge was calibrated using pure nickel (Ni) and setting the binding energy at 0.00 ± 0.02 eV. All XPS spectra were collected at a take-off angle of 90° with spectrometer pass energy of 20 eV. To eliminate any charge induced binding energy shift the XPS spectra reported here are referenced to C 1*s* at 285.0 eV. The core-level spectra are also curve-fitted with a combination of Gaussian and Lorentzian line shapes, using a Shirley-type background subtraction.

## Conclusions

In summary, SnO_2_:Sb thin films were epitaxially deposited, with thickness varied from 3.1 to 188.0 nm by PLD on sapphire 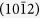 substrates. From the electrical and spectroscopic studies, a size induced MIT and the critical thickness for these films evolving from metallic behavior to insulating nature have been discovered. This approach allows one to examine the size scaling limitation for microelectronic applications. In addition, the quantum confinement effects on the electronic structure of SnO_2_ host and the chemical properties of the impurity element were investigated. A generalized energy band diagram based on the scenario of energetic impurity level pinning and band gap broadening in the doped semiconductor nanostructures was proposed to understand the mechanism of the MIT and the dependence of critical thickness on the impurity concentration in the quantum confined SnO_2_:Sb system.

## Additional Information

**How to cite this article**: Ke, C. *et al.* Thickness-Induced Metal-Insulator Transition in Sb-doped SnO_2_ Ultrathin Films: The Role of Quantum Confinement. *Sci. Rep.* **5**, 17424; doi: 10.1038/srep17424 (2015).

## Supplementary Material

Supplementary Information

## Figures and Tables

**Table 1:** Thickness *t*, valence band maximum (VBM), room temperature electron concentration (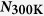), conduction band filling (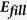), band gap renormalization (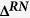), energy band gaps (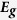), Sb-to-Sn ratio ((Sb^III^ + Sb^V^)/Sn) and Sb(III)-to-Sn ratio (Sb^III^/Sn) for the four SnO_2_:Sb films with different thicknesses.

Sample	A	B	C	D
*t* (nm)	188.0	31.3	7.9	3.1
VBM (eV)	4.07 ± 0.05	4.0 ± 0.05	3.94 ± 0.05	3.77 ± 0.05
*N*_300*K*_ (cm^−3^)	−3.99E20	−3.09E20	−1.55E20	−3.48E17
E_*fill*_ (eV)	0.86	0.75	0.51	~0
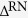 (eV)	0.25	0.19	0.097	~0
E_*g*_ (eV)	3.46 ± 0.05	3.44 ± 0.05	3.53 ± 0.05	~3.77 ± 0.05
(Sb^III^ + Sb^V^)/Sn (%)	5.3	5.8	5.5	5.2
Sb^III^/Sn (%)	0.49	0.51	0.64	1.70
