# Supplementary material for: Thickness-Induced Metal-Insulator Transition in Sb-doped SnO2 Ultrathin Films: The Role of Quantum Confinement
Source: Sci Rep. 2015 Nov 30;5:17424. doi: 10.1038/srep17424 (PMC4663506; doi:10.1038/srep17424)
Supplement: Supplementary Information [file srep17424-s1.pdf]

<Supplementary Information>

Thickness-Induced Metal-Insulator Transition in Sb-doped SnO_2 Ultrathin Films: The Role of Quantum Confinement

Chang Ke, Weiguang Zhu, Zheng Zhang, Eng Soon Tok, Bo Ling, and Jisheng Pan

1. AFM images of the SnO_2:Sb thin films.

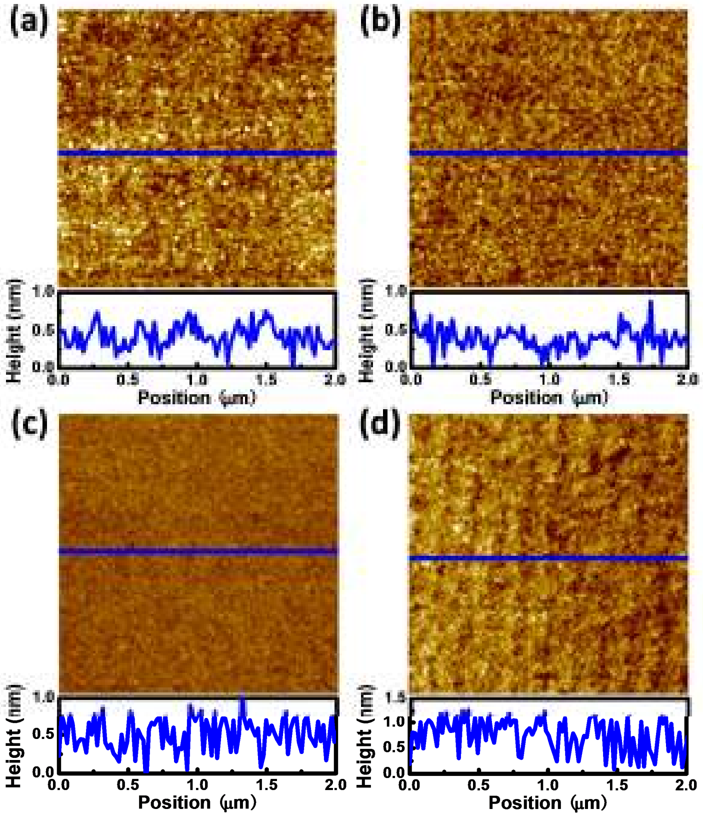

Figure S1. AFM images of the SnO_2:Sb films with thickness of: (a) 3.1 nm, (b) 7.9 nm, (c) 31.3 nm and (d) 188.0 nm, respectively.

The AFM topography images for the 10% doped SnO_2:Sb films were taken (shown in Figure S1). Continuous and smooth surfaces for all the four films were revealed. The RMS roughness values were found to be 0.18 nm, 0.15 nm, 0.23 nm, and 0.31 nm for films with thickness of 3.1 nm, 7.9 nm, 31.3 nm, and 188.0 nm, respectively.

2. XPS survey spectra of the SnO_2:Sb films.

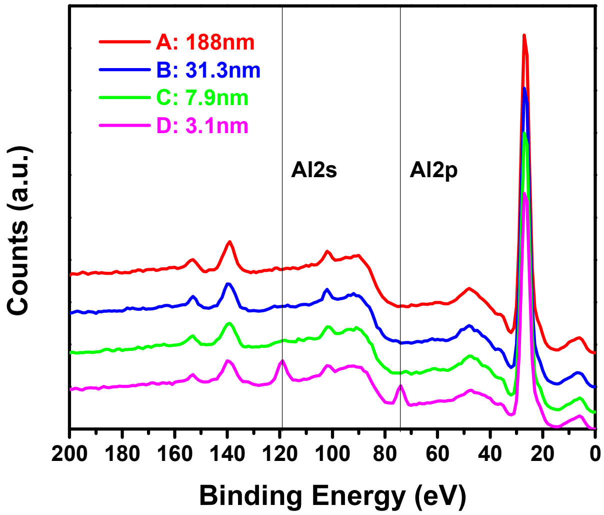

Figure S2. XPS survey spectra of the SnO_2:Sb films with thicknesses: A: 188.0 nm, B: 31.3 nm, C: 7.9 nm and D: 3.1 nm.

XPS survey spectra are collected and shown in the Figure S2. It can be seen that the Al peaks appear in the sample D only, which indicates that the XPS probing depth for our samples is less than 7.9 nm but greater than 3.1 nm.

3. Schematic diagram of conduction band filling and band alignment in XPS VB spectra.

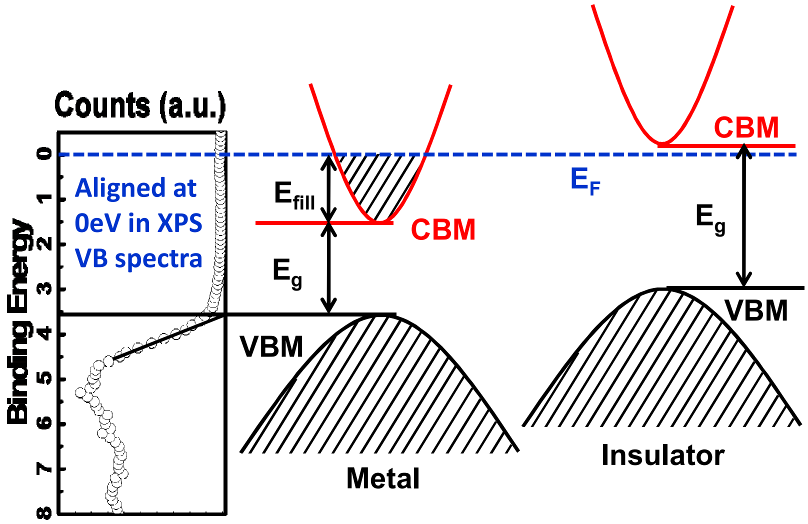

Figure S3. The schematic diagram for the conduction band filling and band alignment in XPS VB measurements for the metallic and insulating SnO_2:Sb thin films.

Figure S3 shows the free electron occupation in conduction band and the band alignment in XPS VB measurements. According to this diagram, the energy band gaps (E_g) for the metallic SnO_2:Sb thin films (samples A to C) can be calculated by subtracting the conduction band filling (E_{fill}) from the VBM. As for the sample D, in which there is no conduction band occupation, we approximate the VBM in VB spectrum to the value of E_g, due to its n-type semiconducting properties revealed in hall measurements.

4. XRD spectra of the SnO_2:Sb thin films.

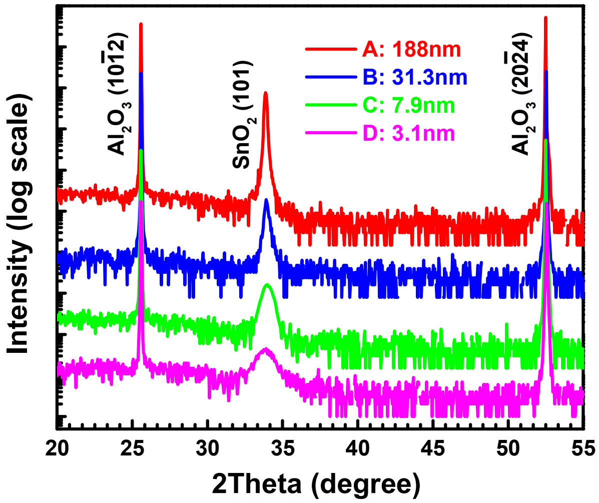

Figure S4. XRD spectra of the SnO_2:Sb films with thicknesses: A: 188.0 nm, B: 31.3 nm, C: 7.9 nm and D: 3.1 nm.

Figure S4 shows the stacked symmetric 2θ-ω scans across the Al_2O_3 $(10\bar{1}2)$, SnO_2 (101) and Al_2O_3 $(20\bar{2}4)$ reflections for SnO_2:Sb films with different thickness. It can be seen that all the films are (101) orientated. The out-of-plane epitaxial relationship between the films and substrate is SnO_2 (101) // Al_2O_3 $(10\bar{1}2)$. There is no prominent SnO_2 (101) peak shift can be seen in the Figure S1. Quantified SnO_2 (101) peak positions and calculated out-of-plane lattice distances are summarized in the Table S1. It is clear that the 2θ and d_{101} do not show thickness dependence, which implies the strain in the samples has been relaxed.

Table S1. Thickness t, (101) peak position (2θ) and calculated (101) plane distance (d_{101}) for the four SnO_2:Sb films with different thicknesses.

Sample	A	B	C	D
t (nm)	188.0	31.3	7.9	3.1
2θ (°)	33.86 ± 0.04	33.90 ± 0.04	33.90 ± 0.04	33.82 ± 0.04
d_{101} (Å)	2.647 ± 0.003	2.644 ± 0.003	2.644 ± 0.003	2.650 ± 0.003

5. Surface of high temperature treated Al_2O_3 $(10\bar{1}2)$ substrate.

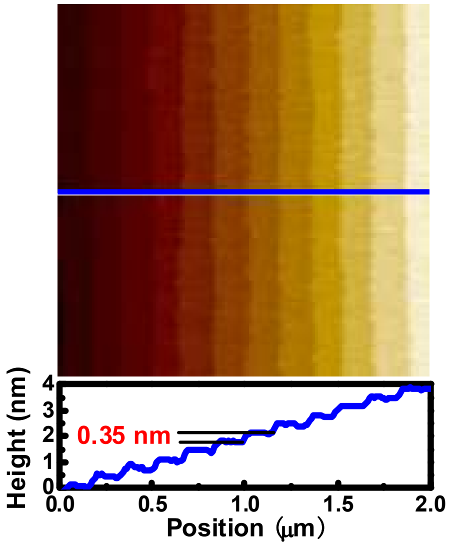

Figure S5. Atomic Force Microscopy (AFM) image of high temperature treated Al_2O_3 $(10\bar{1}2)$ substrate ($2 \times 2\ \mu m^2$). The vertical profile was taken along the line shown in the image, which indicates an atomically flat surface with uniform atomic steps. The observed step height is 0.35 nm, which corresponds to the interplanar spacing of $(10\bar{1}2)$ planes.

The as received Al_2O_3 $(10\bar{1}2)$ substrates were annealed in oxygen ambient at temperature of 1400 °C to achieve the atomically smooth surface (shown in Figure S5) and assist the epitaxial growth of SnO_2:Sb thin films.
